# In-vitro external fixation pin-site model proof of concept: A novel approach to studying wound healing in transcutaneous implants

**DOI:** 10.1177/09544119241234154

**Published:** 2024-03-03

**Authors:** Blake McCall, Karan Rana, Kate Sugden, Sarah Junaid

**Affiliations:** 1 Biomedical Engineering Research Group, School of Engineering and Applied Science, Aston University, Birmingham, UK; 2 Aston Research Centre for Healthy Ageing, School of Life and Health Science, Aston University, Birmingham, UK; 3 Aston Institute of Photonics Technology, College of Engineering and Physical Sciences, Aston University, Birmingham, UK

**Keywords:** External fracture fixation, pin-site infection, human skin equivalent

## Abstract

External fixation is an essential surgical technique for treating trauma, limb lengthening and deformity correction, however infection is common, with infection rates ranging from 4.5 to 100% of cases. Throughout the literature researchers and clinicians have highlighted a relationship between excessive movement of the pin and skin and an increase in the patient’s risk of infection, however, currently no studies have addressed this role of pin-movement on pin-site wounds. This preliminary study describes a novel in vitro pin-site model, developed using a full-thickness human skin equivalent (HSE) model in conjunction with a bespoke mechanical system which simulates pin-movement. The effect of pin-movement on the wound healing response of the skin equivalents was assessed by measuring the expression of pro-inflammatory cytokines. Six human skin equivalent models were divided into three test groups: no pin as the control, static pin-site wound and dynamic pin-site wound (*n* = 3). On day 3 concentrations of IL-1α and IL-8 showed a significant increase compared to the control when a static fixation pin was implanted into the skin equivalent (*p* < 0.05) and (*p* < 0.005) respectively. Levels of IL-1α and IL-8 increased further in the dynamic sample compared to the static sample (*p* < 0.05) and (*p* < 0.0005). This study demonstrates for the first time the application of HSE model to study external-fixation pin-movement in vitro. The results of this study demonstrated pin-movement has a negative effect on soft-tissue wound-healing, supporting the anecdotal evidence reported in the literature, however further analysis of wound heading would be required to verify this hypothesis.

## Introduction

External fixation is a valuable surgical technique for treating open fractures, limb lengthening and deformity correction procedures. External fixation consists of a frame on to which pins are clamped; the pins then pass through the skin to contact the fractured bones in order to support the bones during healing.^1^ Long-term percutaneous implants such as external fixation pins act as an entry point for pathogens which can lead to infection.^2,3^ As a result, pin-site infection rates reported in the literature range from 4.5 to 100%.^4–7^

Wound healing plays a significant part in the development of healthy pin-site wounds. If the wound healing process is impaired, the opportunity for infectious bacteria to enter the host and cause infection is prolonged. Although the presence of a fixation pin prevents the wound from fully closing, therefore the aim of wound healing in external fixation is the development of a collagen shell around the pin which isolates the pin-site wound from the host therefore preventing pathogens from entering the wound.^8–11^

During rehabilitation, early weight bearing is often encouraged, as it permits micro-motion across the fracture, which is known to stimulate osteogenesis^12^ and improve the rate of fracture healing.^13^ However, it is hypothesised that this movement disrupts the development of the soft tissue barrier around the pin, leading to a greater incidence of infection. Throughout the literature, clinicians and researchers have highlighted this relationship, often stating that movement between the pin and skin is a major factor in determining whether a pin-site will become infected.^14–20^

Despite the lack of existing research regarding the process of wound healing in external fixation, several studies have investigated infections in pin-site wounds.^21,22^ The majority of these studies have adopted animal models, which come with their own intrinsic limitations, most significantly a lack of relevance to human physiology. In order to overcome these limitations, in vitro human skin models (HSE) have been developed. HSEs were originally designed with both dermal and epidermal tissue for grafting onto patients suffering from full-thickness skin loss. In recent decades HSE’s have developed in complexity so that they accurately replicate the biological structure human skin and have therefore been used to study various aspects of skin biology, such as skin contraction,^23–25^ skin diseases^26^ and wound healing.^27^

Currently, no attempt has been made to develop an in vitro pin-site model for external fixation, however, several studies have used HSEs to characterise the skin-implant interface in similar percutaneous implants such as dental implants, catheters, glucose sensors, intramedullary prosthesis and feeding tubes. Similarly to pin-site wounds, the wounds these devices create are highly susceptible to infection, often due to the earlier mentioned micro-trauma that disrupts the interface between the skin and biomaterial.^28^ Chai et al.^29^ developed a novel in vitro three-dimensional oral transmucosal model (3D OMM) by culturing an acellular dermal explant scaffold with human keratinocytes and gingival fibroblasts to investigate the implant-soft tissue interface in dental implants. They described the 3D OMM as forming a peri-implant-like epithelium attachment to the titanium sample, similar to the epithelium structure seen in animal models, demonstrating that it is possible to use HSE models to study percutaneous implants.

More recently, Bolle et al.^30^ developed an in vitro HSE model to study the role of skin integration around drivelines on bacterial infection. Bolle demonstrated that bacterial migration along the drive line surface occurs in micro-gaps caused by insufficient skin integration between the driveline and surrounding skin. The results of this study supported current knowledge on driveline infections derived from in vitro agar models^31^ ex vivo analysis of explanted drivelines^32^ and in vivo rodent models,^33^ demonstrating the physiological relevance of in vitro HSE studies. However, the authors suggested the model could be further improved though the addition of movement of the driveline to mimic patient breathing and other motion which may disrupt wound healing around the pin.^30^

Wound healing of an in vitro HSEs can be monitored through a number of biomarkers. Inflammatory cytokines released from platelets, neutrophils, macrophages and epidermal cells are up-regulated during the inflammatory phase to act as signalling molecules between cells to initiate various wound healing processes such as cellular growth, proliferation, differentiation and re-epithelialisation.^34^ By using ELISA tests to measure the concentration of these cytokines expressed by the HSEs under various treatments, the efficacy of that treatment can be assessed. Cytokine levels of IL-1α, IL-6 and TNF-α have been found to be significantly higher in non-healing wounds compared to that of healing wounds,^35^ with concentrations of IL-1α almost 3.5x greater in non-healing chronic leg ulcers compared to that of healing wounds.^36^

Skin movement varies widely and will have a direct effect on pin site movement and wound healing, which merits further study. The actual degree of pin movement is not known, however studies on the discrepancy between skeletal movement and soft tissue translations, known as the soft tissue artefact (STA), can be used to estimate the magnitude and frequency of such movement. Many attempts have been made at categorising these artefacts by using surface markers along with fluoroscopy^37–40^ invasive pins such as intra-cortical pins^41–43^ percutaneous trackers^44^ and external fixators.^45^ Several studies have observed a greater magnitude of STA in the shank compared to that of the thigh^46–48^ with many studies showing that skin mounted markers can exhibit displacements relative to the underlying bone of anywhere between 0.7 and 10.9 mm.^49,50^

The aim of this study was to develop a novel in vitro pin-site model consisting of a mechanical pin-motion system and bioreactor for culturing HSE models, allowing for regular sampling of the culture medium for cytokine analysis. We hypothesis that the presence of the pin with have a negative effect on wound healing characterised by an increase in cytokine production, which will be exacerbated when movement is applied to the pin.

## Methods

### In-house built pin-movement machine

**Figure 1.**
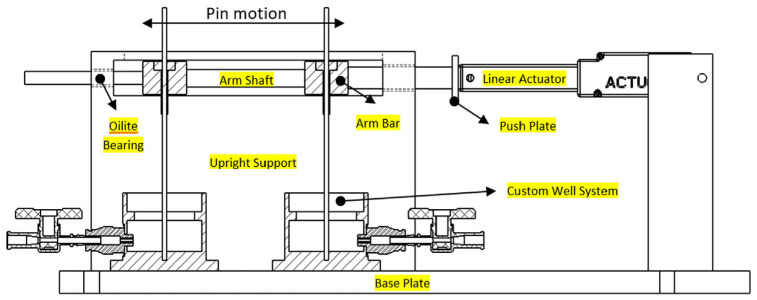
Drawing of final pin machine design with main components annotated.

**Figure 2.**
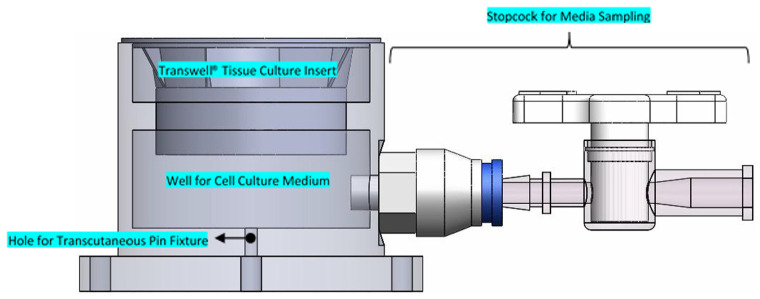
Model of custom well system highlighting key design features – the design of the well ensures a clearance fit between the well and Transwell® insert, allowing easy removal of the Transwell® insert while preventing lateral movement of the insert to ensure any pin movement is translated directly to the soft tissue interface. A well below the Transwell® insert allows for addition of cell culture media to maintain an air-liquid interface. The stopcock allows access to the media for sampling or the addition of fresh media.

A novel mechanical system was constructed in-house to provide both mechanical movement to fixation pin as well as provide a suitable environment for culturing the skin models (Figure 1). A linear actuator L16-R (Actuonix Motion Devices, Canada) was employed to generate the linear displacement of pins and an Arduino ATMEGA 2560 microcontroller (Arduino™, Italy) was used to control the magnitude and frequency of the linear displacement. The skin models were grown on 24 mm Transwell® inserts, which were placed in a custom-designed aluminium well system and covered in Parafilm™ (Thermo Fisher Scientific, UK) to maintain sterility (Figure 2). The pins were implanted by pushing the pin through the HSE into a concentric hole at the base of the well. A stopcock at the base of the well allowed for the sampling of media for analysis or the addition of fresh medium to maintain an air-liquid interface.

### Cell culture

Primary human dermal fibroblasts (HDF) (PromoCell, Delaware, USA) and normal primary human epidermal keratinocytes (NHEK) (PromoCell, Delaware, USA) were used for the culture of the HSE models. Both cell types were cultured up to a maximum of 15 population doublings as per the manufacturer’s recommendations.

HDF cells were maintained in serum-free, low glucose Dulbecco’s Modified Eagle Medium (DMEM) (Thermo Fisher Scientific, UK) supplemented with 10% (v/v) foetal bovine serum (FBS) (Thermo Fisher Scientific, UK), 8mM HEPES (Sigma Aldrich, UK), 10,000 U/mL penicillin and 10,000 µg/mL streptomycin (Thermo Fisher Scientific, UK). The keratinocytes were cultured in serum-free keratinocyte basal medium with additional Growth Medium 2 Supplement Pack (PromoCell, UK)

### Human skin equivalent model

The HSE models were prepared using a previously reported method.^51^ Details on various components and their volumes used to construct the collagen matrixes as well as all the necessary cell culture mediums can be found in this paper. The HSEs were cultured on 0.4 μm permeable Transwell® inserts with a 4.67 cm^2^ surface area (Corning, USA). Before seeding the cells, the Transwell® inserts were coated with 1 mL of acellular collagen mixture, which was allowed to fully gel at room temperate. 18mL of cellular collagen matrix was then prepared, containing 1.65 mL of HDF suspended in FGM at a cell density of 3 × 10^5^ cells/mL. An aliquot of 3 mL of the cellular matrix was added to each Transwell® insert, and the 6-well plate was immediately returned to the incubator for 30–60 min at 37°C and 5 % CO_2_. Once the mixture had completely gelled and turned pink, the gels were fed with 4 mL of FGM, adding 3 mL to the outside of the insert and 1 mL directly on-top of the insert. After 1 week the dermal component was fully matured and ready to be seeded with NHEK. NHEK were re-suspended to a cell density of 3 × 10^6^ cells/mL and an aliquot of 50 µL of the NHEK cell suspension was then transferred onto each Transwell® insert and left for 15 min without moving to allow the cells to attach to the surface of the dermal model. After which the 6-well tray was returned to the incubator for 1 hour at 37°C and 5% CO_2_ without any additional medium, allowing the NHEK to fully adhere to the dermal model. Once the cells had fully adhered 4 mL of KGM was added to the outsides of each well and 1 mL added on top. After seeding keratinocytes onto the dermal component, the skin models were fed with epidermalisation media for 6 days followed by cornification media for 14 days, ensuring the HSE model was maintained at an air-liquid interface.

### Histological analysis with H+E staining

Once fully mature, the HSE’s were carefully separated from the Transwell® membrane using a surgical scalpel. The excised tissue was then placed in a plastic tissue cassette and immersed in 10% formalin (v/v) (Thermo Fisher Scientific, USA) for 1 h. Tissues were then dehydrating by immersion in ethanol gradations and embedded in paraffin wax. Thin sections (5 µm) were prepared using a microtome and de-waxed with a series of xylene and alcohol solutions. De-waxed samples were then stained in Haematoxylin and Eosin (Thermo fisher scientific, USA) and imaged at 40× magnification (Figure 4).

### IL-6/IL-8 release analysis

After 24, 48 and 72 h of exposure to each treatment condition the cell-culture supernatants were collected in microfuge tubes and cleared of any cells by centrifuging at 1000*g* for 5 min. The supernatants were then analysed for Tumour Necrosis Factor-alpha (TNF-α), Interleukin 1 alpha (IL-1α and Interleukin 8 (IL-8) pro-inflammatory mediators via enzyme-linked immunosorbent assay (ELISA). Commercially available e-Bioscience ELISA kits (San Diego, USA) were used for this purpose. All assays were performed according to the manufacturer’s instructions, including the assay protocol and preparation of reagents.

### Experimental design

**Figure 3.**
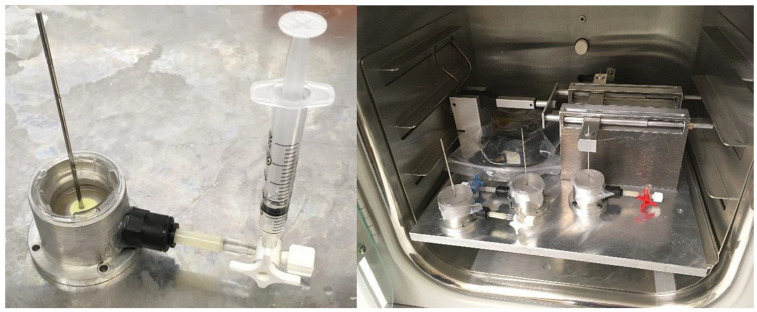
Images of pin machine during incubation and sampling of media from custom well. Skin models were placed in the aluminium wells and the skin implanted with a fixation wire.

This study contained three experimental groups; the ‘dynamic’ group consisted of a HSE model implanted with a 1.8 mm fixation pin. Movement was applied to the pin at an amplitude of 1mm simulating micro-motions of the pin which is a known precursor to infection in percutaneous devices^28^ and a frequency of 1 Hz replicating average gait cycle frequency^52^ (*n* = 3). The ‘static’ group also consisted of a HSE model implanted with a fixation pin, however no movement was applied to this pin (*n* = 3). The negative ‘control’ group consisted of a HSE model only with no fixation pin implanted (*n* = 3). After implanting the pin, each sample was fed with 10 mL of cornification medium to achieve an air-liquid interface and covered with a layer of Opsite (Smith & Nephew, USA) to maintain a sterile environment. The mechanical pin machine was battery powered and placed in an incubator at 37°C and 5% CO_2_. At 24, 48 and 72-h samples of the media from each pin-site model were taken. A syringe was attached to the stopcock of the aluminium well and a 1 mL sample of the culture medium was taken and transferred to a 1 mL Eppendorf tube and frozen at −80°C until ELISA analysis was conducted. After sampling, 1 mL of cornification media was replaced into each well (Figure 3).

**Figure 4.**
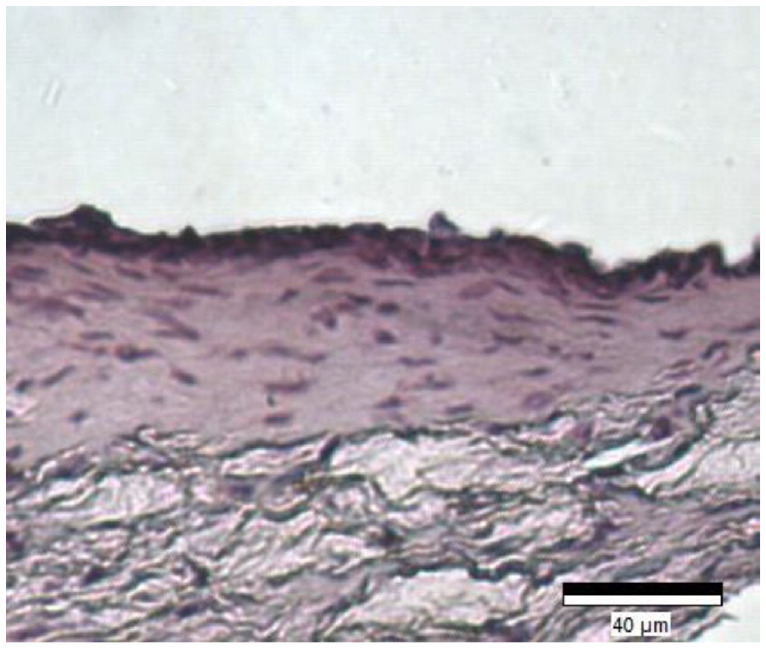
Five micrometre sections of the collagen-based full-thickness human skin equivalent stained with H&E. Distinct dermal and epidermal layers can be seen. Dermal layer consists of long collagen fibres (pink) impregnated by fibroblasts (dark purple/blue) and epidermal layer shows several layers of keratinocyte cells (dark purple/blue).

#### Statistics

The data collected throughout this study were analysed for statistical significance using a two-tailed unpaired Student’s *t*-test when comparing the difference between two group means. When comparing groups split on two independent variables a two-way analysis of variance was used (*p* = 0.05).

## Results

### Histological analysis of dermal equivalent models

#### Cytokine expression over 72 h

**Figure 5.**
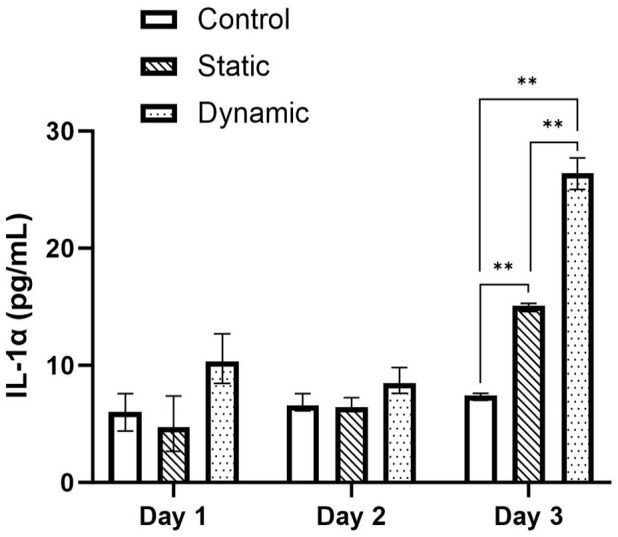
Concentrations (+/− standard deviation) of IL-1α cytokine on days 1, 2 and 3 for the control, static and dynamic samples (*n* = 3). On day 3 cytokine concentration increased significantly between control, static and pin samples (*p* < 0.05).

**Figure 6.**
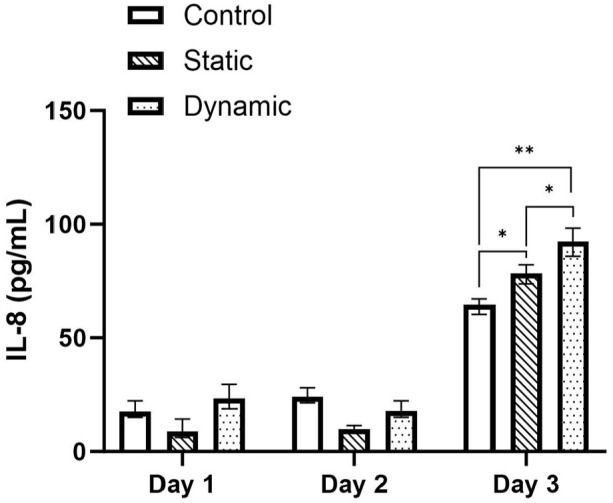
Concentrations (+/− standard deviation) of IL-8 cytokine on days 1, 2 and 3 for the control, static and dynamic samples (*n* = 3). On day 3 cytokine concentration increased significantly between control, static and pin Samples (*p* < 0.05).

**Figure 7.**
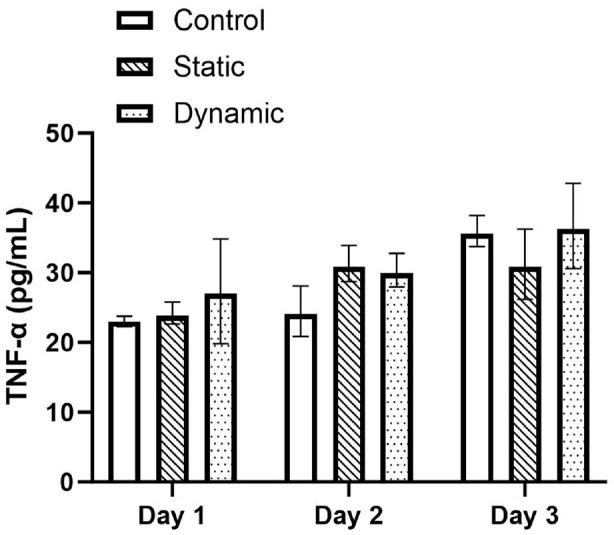
Concentrations (+/− standard deviation) of TNF-α cytokine on days 1, 2 and 3 for the control, static and dynamic samples (*n* = 3). No significant difference was observed between the control, static and dynamic samples across all days.

Cytokine concentration of the media was calculated from the absorbance values using the standard curves of each ELISA assay. Figures 5 to 7 presents the cytokine concentrations for IL-1α, TNF-α and IL-8 for the control, static and dynamic pin samples on day 1, 2 and 3 of the experimental periods respectively. No significant difference was observed between day 1 and 2 for all samples and cytokines tested. However, the expression of IL-1α increased in the static and dynamic samples significantly from day 1 to day 3 (*p* < 0.05) (Figure 5). IL-1α expression increased by 103.5% in the static pin sample compared to control, which increased a further 75.0% in the dynamic pin sample compared to static (Control: 7.41 ± 0.187 pg/mL; Static: 15.08 ± 0.169 pg/mL; Dynamic: 26.39 ± 1.35 pg/mL). Similarly, the expression of IL-8 significantly increased in the static and dynamic samples from day 1 to day 3 (*p* < 0.05) (Figure 6). An increase of 21.4% was observed between the control and static samples (*p* < 0.05) which increased by a further 42.9% between the control and dynamic samples (*p* < 0.05). There was also a significant difference in IL-8 expression of 17.7% between the static and dynamic samples (*p* < 0.05). (Control: 64.59 ± 0.096 pg/mL; Static: 78.41 ± 5.31 pg/mL; Dynamic: 92.33 ± 3.68 pg/mL). However, although the expression of TNF-α appeared to increase between days 1 and 3, only the control sample showed a significant difference (*p* < 0.05) (Figure 7). On day 3 no significant difference was observed between the control, static and dynamic samples.

## Discussion

Pin-site infection in external fixation is extremely common.^4–7^ Many clinicians have suggested that pin movement is a major contributor to the high incidence of pin-site infection due to movement disrupting the wound healing around the pin.^14–20^

Multiple approaches have been attempted to prevent or minimise pin-site infection with varying degrees of success, including compressive dressings or the use of advanced pin materials, coatings or topical ointments.^53–59^

The majority of these studies have been in vivo or animal studies, which are costly, difficult to control, lack physiological relevant or have ethical implications. The development of a realistic pin-site model would allow for high throughput testing to better understand the mechanisms of pin-site infection and study the efficacy of these treatments on a larger scale.

Therefore, primary aim of this research was to develop an in vitro external fixation pin-site model to study the effect of pin movement on the mechanisms of wound healing. A custom mechanical loading system was developed which applied linear movement to the pins as well as providing a suitable environment for the culture of the HSE models. The HSE models themselves were cultured based off of an existing protocol,^51^ and were confirmed via histology to have similar morphology to that of native human skin. We hypothesised that the presence of an implanted fixation pin into the HSE would have a negative effect on wound healing, characterised by an increased production of IL-1α, IL-8 and TNF-α cytokines compared to the negative control, which would increase further when movement was applied to the pin.

On day-3 of the experiment, concentrations of IL-1α and IL-8 showed a significant increase in the static pin samples compared to the control. Furthermore, a significant increase in IL-1α and IL-8 was also observed in the dynamic pin sample compared to the static pin sample. This result provides some support to our hypothesis, as well as some validity to the use of the HSEs as a wound model. However, no significant difference in TNF-α was observed between all samples tested. Suggesting that TNF-α is a poor measure of wound healing response in HSE models. These results reflect those reported in the literature, for example, Spiekstra et al.^60^ measured the cytokine levels of ex vivo skin and observed IL-8 and IL-1α levels of 19.64 ± 13.51 and 22 ± 15 pg/mL respectively, while no levels of TNF-α were detected. While the greatest increase in cytokine expression between the control, static and dynamic samples on day 3 were observed for IL-1α, the concentration of IL-8 was much higher in all samples tested. IL-8 is known to influence the wound healing processes of proliferation and migration in keratinocytes, as well as reducing fibroblast-associated contraction,^61^ an effect that can be observed in the contraction of the HSE model during the first week of culture.

This was a proof-of-concept study and as such there were several limitations. Firstly, despite playing an important role in wound healing, analysis of cytokines alone is not sufficient in determining the effect of a given treatment on wound healing. In future studies, additional analysis should be conducted to provide further support to our hypothesis. For example, post-experimental histological analysis may be used to visualise cell growth around the pin-site or additional wound healing biomarkers such as matrix metalloproteinases (MMP) and tissue inhibitors of metalloproteinases (TIMPs) may be measured. Secondly, on day 1 of the experiment, all cytokine concentrations measured were close to the lower limit of detection of the ELISA assay. This was likely due to the design of the tissue culture well, which required 15mL of medium to maintain an air-liquid interface across the HSE, therefore any cytokines expressed by the skin equivalent on day 1 were highly diluted in the media. This issue could be rectified by a redesign of the well to minimise the volume of media required to achieve an air-liquid interface and therefore minimise the dilution of cytokines in the media. Finally, no existing study has characterised the degree of pin movement experienced across the pin-site. Therefore, we utilised soft-tissue artefact translations obtained from measurements between skeletal trackers and percutaneous implants. As a result, we only tested the effect of linear movement across the pin-site when we would expect to see both shearing of the pin along the soft tissue resulting from changes in muscle volume due to the host’s muscle contractions as well as rotational movement generated from torsional loading across the frame. Our research would benefit from a clinical study to better understand the translations of the pin to develop a standardised testing protocol for movement in external fixation.

The authors would progress the described methodology by testing wound healing within animal models as an appropriate next step, in a bid to capture the healing process in vivo. However, the work of Mertsching et al.,^62^ demonstrates that the 3-D skin equivalent closely resembles natural skin from a physiological perspective, making it a viable substitute for in vivo research. Additionally, this approach offers both ethical and economic advantages, leading to a shift in testing strategies within the Pharmaceutical Industry away from animal testing. A key factor involved in keeping a wound from healing properly includes an increased and/or prolonged inflammatory reaction. The methodology employed within this study is consistent with the latest research. Szilagyi et al.,^63^ measured circulating concentrations of MMP-1, IL-1α, IL-6 and IL-8 following ultraviolet and Wi-Fi radiation in a 3D human skin model. Further research ought to employ a more molecular methodology and involve quantifying protein expression of pro-inflammatory markers through western blotting and subsequently, support this data with gene expression of the same markers, via PCR. Real-time analysis of wound healing, using hyperspectral imaging has been described by Wahabzada et al.,^64^ and serves to interpret wound healing using an efficient approach for unsupervised classification of wound tissue based on hierarchical decomposition according to archetypal data points. Finally, despite the HSE models being grown in quintuplets and experiments completed at a n of 3, as per previous published studies,^65^ the authors would have liked to repeat the experiments on a few more occasions.

Our in vitro pin-site model and incubator pin-machine provides the framework for future studies to investigate the efficacy of pin-site care treatments aimed at minimising pin movement. To date, such a physiologically relevant model has not been achieved previously and offers an important tool in studying treatment efficacy in a controlled way. Additionally, the design of our in vitro model is such that other percutaneous devices could also be studied. Devices such as catheters, cochlear implants, dental implants, and prosthetic limb anchors and drivelines all suffer from high rates of infection of which could be minimised through adequate wound healing. Our model could also be developed further to incorporate infectious bacteria to study the effect of bacterial colonisation on the wound healing response. Several studies have investigated the effect of bacteria colonisation to HSE models,^30,66–68^ this approach could be used develop our model further, in order to study the relationship between wound healing, mechanical motion and pin-site infection in detail.

## Conclusions

This study demonstrates for the first time using a validated in vitro model that mechanical motion of an external fixation pin has a significant effect on the wound healing of soft tissue around the pin. A novel validated application for HSE has been presented which investigated the biological response to mechanical stimuli. The model and testing system can potentially be used to investigate the interaction between pin-site healing, mechanical motion and wound response on a fundamental level as well as testing the efficacy of novel treatments. The results in this study demonstrate the effect of pin presence on wound healing response, which is exacerbated when movement is applied to the pins. The findings corroborate with the anecdotal evidence reported throughout the literature that minimising pin movement may be a key factor in minimising pin-site infection in external fixation. However further wound healing analysis would be required to verify this hypothesis.
